# Using an Intersectional Approach to Explore the Lived Mental Health Experiences of Traveller Men Affected by Suicide in Ireland

**DOI:** 10.1177/15579883231189063

**Published:** 2023-09-09

**Authors:** Noel Richardson, Karolyn McDonnell, Paula Carroll, Shane O’Donnell

**Affiliations:** 1 South East Technological University, Carlow, Ireland

**Keywords:** suicide, ethnic minority, intersectionality, Traveller masculinities

## Abstract

Rates of suicide are seven times higher among Traveller men, an Indigenous ethnic minority group in Ireland, compared with non-Traveller men. Several factors are implicated, including racism, social exclusion, discrimination, inadequate accommodation, unemployment, and lower educational attainment. Systemic and cultural barriers inhibit Traveller men from seeking support. This study addresses a gap in the literature by exploring the lived mental health experiences of Traveller men affected by suicide. Semi-structured interviews (*n* = 13; aged 19–50) were conducted with Traveller men affected by suicide. Interviews were recorded and transcribed verbatim. Thematic content analysis was used to analyze the data, which yielded three broad themes. Theme 1, “key determinants of Traveller men’s mental health,” describes the impact on Traveller men of issues relating to accommodation/homelessness, education, and unemployment, as well as frequent exposure to prejudice, discrimination, and racism. Theme 2, “contemporary Traveller masculinities,” considers how Traveller masculinities were shaped by a patrilineal tradition and by historical/ongoing tensions related to their ethnicity. Theme 3, “navigating support seeking and coping with distress,” encapsulates both resistant and proactive approaches used by participants to manage their mental health. The intersection of structural inequalities, internalized racism, Traveller masculinities, and strong historical associations between stigma and mental health/suicide within the Traveller community lies at the heart of the heavy burden of suicide carried by Traveller men. Findings provide a deeper understanding of the sources of distress and pathways to resilience/recovery among Traveller men affected by suicide and can inform the development of more gender- and culturally appropriate suicide prevention interventions.

## Introduction

### Travellers: The “Other” of Irish Ethnic Identity

Irish Travellers are an Indigenous ethnic minority group with a shared history, culture, and traditions, including, historically, a nomadic way of life (Equal Status Act, 2000; Hayes, 2006). They account for just 0.7% of the general population in Ireland but experience a disproportionate burden of ill health. This is particularly pronounced in relation to mental ill-health where suicide accounts for 11% of all deaths among Travellers. The Traveller male suicide rate is seven times higher than the non-Traveller male population in Ireland and presentation at emergency services with suicide ideation is four times greater (All-Ireland Traveller Health Study [AITHS], 2010; Kavalidou et al., 2022). These comparatively higher rates of suicidal behavior among Traveller men are mirrored in other Indigenous communities such as those in Australia (Australian Institute of Health and Welfare [AIHW], 2022), Canada (Hajizadeh et al., 2021; Kumar & Tjepkema, 2019), and New Zealand (Ministry of Health [MOH]—Manatū Hauora, 2021). Depression and anxiety among Irish Travellers have been reported to be two and three times higher compared with a non-Traveller population (Parry et al., 2007). Travellers have been identified as a “priority group” at a suicide prevention policy level in Ireland (Department of Health, 2015).

Systemic racism, discrimination, social exclusion, material deprivation, and inequalities in both access to and outcomes in education, employment, and health have all been implicated in these high rates of suicide behavior. For example, based on secondary analysis of Census 2011 data, Watson et al. (2017) revealed that Travellers are much more likely to have left school at an early age, with 28% of Travellers above 25 years having left before the age of 13, compared with 1% of non-Travellers. The same report found that just 8% of Travellers had completed education to “Leaving Certificate”^1^ compared with 73% of non-Travellers, whereas only 1% of Travellers aged 25 to 64 years had a college degree compared with 30% of non-Travellers. Similarly, only 11% of Travellers were found to be in employment, in comparison with 66% of non-Travellers. Among those aged 25 to 64 years, the unemployment rate was 82% for Travellers in Census 2011, compared with 17% for non-Travellers. Furthermore, Travellers were significantly more likely to be homeless (39% vs. 0.2%) or to be living in accommodation with more than one person per room (56% in comparison with 8% of non-Travellers; Watson et al., 2017). The AITHS (2010) found that Travellers were five times more likely to be in prison than non-Travellers. In comparison with the non-Traveller population, Travellers are significantly more likely to experience discrimination in accessing paid work (10 times higher), accommodation (10 times higher), and private services such as shops, pubs, and restaurants (22 times higher) (McGinnity et al., 2017). It is against this wider backdrop of systemic racism, marginalization from key societal institutions, and acculturation that Hayes (2006) describes Travellers as the “other” of Irish ethnic identity. This is reflected in a qualitative study which reported that Travellers feel disconnected, unwanted, and hopeless and experience shame about their cultural identity (Villani & Barry, 2021).

### Factors Underpinning the Increased Suicide Risk Among Traveller Men

The normlessness and disturbance imposed by loss of tradition and culture can disrupt Travellers’s sense of identity within their community as well as within the self, leading to feelings of powerlessness and increased risk of suicide (Hodwitz & Frey, 2016; McKey et al., 2022; O’Donnell & Richardson, 2020). Bereavement has also been identified as a significant risk factor for suicide among the Traveller community (AITHS, 2010). All of these issues are considered risk factors for suicide more generally (World Health Organization [WHO], 2014) and can be compounded by male-specific risk factors such as use of more lethal means and a reticence to seek help for mental health issues. Applying Connell’s (1995) frame to the risk factors most often implicated in male suicide, unemployment and financial insecurity can disrupt men’s sense of role and status due to the centrality of the provider role in the construction of the masculine self (O’Donnell & Richardson, 2020; Payne et al., 2008). Men’s use of more lethal means is proposed to reflect a hypermasculinity of sorts where lethality is associated with mastery and where “failure” is less acceptable (Payne et al., 2008). Men can be reticent about seeking support for mental health problems in a bid to remain stoic and self-reliant and cope through more “male-acceptable” outlets such as alcohol and substance misuse (Galdas et al., 2005; Pirkis et al., 2017; Richardson et al., 2013). These maladaptive coping mechanisms can escalate to what Brownhill et al. (2005) describe as the “big build” and can ultimately contribute to suicidal behavior. Although previous studies have suggested that Traveller men’s experience of structural inequalities, racism, marginalization, and discrimination perpetuates the construction of more traditional masculinities and lead to increased suicide risk (Cush et al., 2020; O’Donnell & Richardson, 2020), more research is needed to explore how these structural barriers interact with gendered and cultural expectations among Traveller men to impact their mental health.

Several studies have documented wide-ranging impediments to Traveller engagement with health services such as not having a consistent accommodation status leading to difficulties with registration and sustained engagement with services, racism, and discrimination from health care professionals, literacy issues in completing appropriate documents, and an overall lack of culturally appropriate services (Brack & Monaghan, 2007; Cush et al., 2020; European Union Agency for Fundamental Rights (FRA), 2019; McFadden et al., 2018). These low levels of Traveller engagement with mental health services has been described as “particularly concerning” (Traveller Health Unit, 2019, p. 17) and may further compound mental health issues among this population. Cush et al. (2020) refer to this broad spectrum of inequalities as “accumulated exclusions” which interplay to affect mental health. Despite repeated equality-driven calls for increased rights and respect for the Traveller community (Equal Status Act, 2000; Watson et al. (2017), negative views toward Travellers persist in Irish society (European Union Agency for Fundamental Rights (FRA), 2019). A lack of appropriate Traveller accommodation and criminalization of nomadism—a key aspect of Traveller culture—is felt to contribute to widespread anomie within the Traveller community (O’Shea Brown, 2020). However, in seeking to unravel the factors underpinning Traveller mental ill-health, no study to date has addressed how these wider determinants of health intersect with other axes of inequity (ethnicity, gender, class, etc.) to bring about intersecting systems of oppression and marginalization, particularly among Traveller men.

### Using an Intersectional Approach to Address Suicide Among Traveller Men

Over the past 10 years, scholarship has begun to explore how health and social inequities experienced by boys and men are a consequence of multiple intersecting structural factors. There has been an increasing focus, in particular, on applying an intersectional lens to unravel how mental health experiences are shaped by the unique positioning of individuals within complex social systems of privilege and marginalization (Griffith et al., 2013). Intersectionality seeks to embrace the complexity of how socially prescribed roles and attributes are inherently linked, and that cannot be separated into discrete axes that operate independently or additively (O’Donnell & Richardson, 2020). Rather, it considers simultaneous interactions of social identities, locations, and structures that have a multiplicative effect on social (dis)advantage and that capture overlapping systems of privilege, subordination, and marginalization (Hankivsky, 2013). The focus of this study, therefore, is on social axes typically utilized in intersectionality research (gender and ethnicity) as well as other social categories that have been linked to suicide within the general population but not widely investigated among Traveller men (age, relationship status, occupational status, educational attainment, and accommodation status). The aim of this study was to explore the lived mental health experiences of Traveller men affected by suicide (i.e., those who had in the past reported suicidal thoughts, attempted suicide, or having been bereaved by suicide, excluding within the preceding 3 months) in Ireland. In doing so, the study sought to address two research questions:

**Research Question 1:** What are the sources of psychological distress among Traveller men affected by suicide in Ireland?
**Research Question 2:** What are the barriers and enabling factors to Traveller men seeking help and accessing support during times of psychological distress?

In particular, we adopted an intersectional approach to examine how multiple layers of disadvantage are experienced by Traveller men, with a view to gaining deeper insights into the factors underpinning suicide among this population. This study forms the first phase in the design of a wider therapeutic mental health intervention, using the medium of Digital Storytelling (DST), targeted at Traveller men.

## Method

### Study Design

This exploratory study adopted a qualitative design to meet the study objectives. In devising the research methodology, we considered key contextual factors, including the highly emotive topic of suicide within the Traveller community (AITHS, 2012), the traditional distrust between Travellers and non-Travellers (Hodgins & Fox, 2014), the under-representation of Traveller men in research studies (Bonevski et al., 2014), as well as the imbalanced power relationships entwined in “doing research” with marginalized groups (Brown & Scullion, 2010). Consequently, we enacted two important paradigms of research: (a) working in partnership with nongovernmental organization Pavee Point Traveller and Roma Centre in codesigning and coleading the research study with a commitment to developing lateral research capacities, and (b) utilizing a community-based participatory research (CBPR) design authentically with Traveller Health Workers and participants (Beebeejaun et al., 2014; Hacker, 2013; Israel et al., 2008). Partnership working in research strengthens the impact and reach of studies and is more likely to result in mutually beneficial outcomes and enhanced engagement by the target demographic (Turin et al., 2022). Therefore, the research team engaged closely with and sought to understand the perspectives of the Traveller Health Workers, who were an integral part of developing the topic guide and identifying and recruiting participants to contribute to the study. The study was overseen by an Advisory Group which comprised members from Pavee Point Traveller and Roma Centre and men’s health organizations as well as the Health Service Executive (HSE) in Ireland. Prior to commencing data collection, members of the research team engaged in anti-racism training which helped the team members to reflect on any potential biases, assumptions, or preconceptions about Travellers. All these considerations were deemed crucial in ensuring transparency and trustworthiness in the research design. Funding for the study was provided via a research grant from the National Office for Suicide Prevention (NOSP). The study received Ethical Approval from South East Technological University (SETU) Ethics Committee (Ethical Approval Number 304).

### Participants

Criteria for inclusion in the project were male members of the Traveller community, aged >18 years, who had in the past reported suicidal thoughts, attempted suicide, or having been bereaved by suicide (excluding within the preceding 3 months). Participants were recruited using purposive and snowball sampling techniques. Traveller Health Workers acted as a bridge between the research team and potential research participants, by approaching an initial sample of six Traveller men who met the inclusion criteria but were screened for sensitivity to discussing the issues, appraising them of the purpose and scope of the research, and requesting their participation. These assisted with recruiting additional participants, each of whose suitability was considered by the Traveller Health Workers in terms of sensitivity to discussing the issues. The aim was to gather a diverse sample in terms of age, occupational status, marital status, and so on. Before informed consent was obtained, participants were informed about the scope of the study, the potential risks of participation, and their right to withdraw from the study at any time. The study information sheet was read aloud and participants were afforded as much time as they needed to consider their participation, and to ask questions. Following this, the informed consent sheet was read aloud and participants gave their written consent. Traveller Health Workers were consulted in advance to advise on any literacy issues, but none were encountered.

### Data Collection

Data collection comprised face-to-face individual interviews with 13 Traveller men. We aimed to recruit a diverse sample in terms of age, employment status, accommodation status, educational level, and marital status. Due to the relatively small number of Traveller males in Ireland (*n* = 20,000) and at the request of project partners Pavee Point and the project Advisory Group, we have deliberately excluded specific sample demographics. This was based on assurances given by the project partners to participants regarding anonymity and confidentiality, and concerns, in particular, about preserving the anonymity of research participants. In terms of demographics, participants ranged in age from 18 to 48; their marital status included married/living with a partner, single, and separated/divorced; their employment status included employed, unemployed, and student; their educational status included primary only, secondary, and third level; and their accommodation status included Traveller-specific (halting sites), mainstream, and emergency accommodation.

Semi-structured interviews were conducted in June/July 2021 by members of the research team (K.M.D. and S.O.D.) in community settings that were selected jointly by participants and Traveller Health Workers. The latter were in an adjoining room to offer follow-up support and signposting to appropriate services should the interview triggers any potential distress for participants. Participants were also provided with contact details for support services and encouraged to access them if needed. Both interviewers were trained in “ASIST” (Applied Suicide Intervention Skills Training) and equipped with the skills to offer support to an interviewee who may have shown any signs of potential risk. After each interview, the interviewer had a debriefing session with the principal investigator. In the event of any concerns being raised, follow-up contact was made with the appropriate Traveller Health Worker to investigate such concerns. The topic guide was developed based on the relevant literature and in consultation with Traveller Health Workers and the Advisory Group. Open-ended questions explored conceptualizations of mental health and well-being, sources of psychological distress, and support-seeking behaviors. In keeping with previous research (O’Donnell & Richardson, 2020) by the lead author for this study (N.R.), the meaning of the term “psychological distress” was grounded in participants’ personal experiences and incorporated a broad range of distress, including the potential for discussions around past suicidal behavior. Close attention was paid to how masculinity, age, and various other sociodemographic characteristics played a role in these mental health experiences. Data collection ceased once no new information was observed during data collection. Field notes and a reflective journal were used to record observations, to maximize transparency in data collection, and to contextualize these verbal accounts during transcription and data analysis.

### Data Analysis

Inductive thematic analysis was utilized to analyze the data (Braun & Clarke, 2021). This approach firmly situates the researcher at the center of the qualitative process. Interviews were digitally recorded and transcribed verbatim. Transcripts were independently, line by line coded by the first and second authors (N.R. and K.M.D.). The research team then cross-checked coding strategies, negotiated interpretations, collapsed codes, and agreed on a cumulative or “master” code list. Codes were sorted into potential themes and aligned with the associated data extract. Guided by the principal research questions, the authors worked with drafted sections for each code to further identity and refine thematic labels. Through the writing up of this article, consensus was reached among all authors about theme names and content upon which analysis was finalized. Theme memos and concept maps were utilized to track relationships between codes and categories over the analysis process. Pseudonyms are used for direct quotes. Due to the difficulty in following up with participants, it was beyond the scope of this study to conduct validity checks. However, the research team actively engaged with Traveller representatives from the Advisory Group (including two male Traveller Health Workers) at particular points throughout data analysis and report writing to apply a more nuanced and culturally sensitized lens to data analysis. Adopting a comprehensive and rigorous audit trail of the research process helped maximize transparency and trustworthiness in data analysis.

### Key Terms

“Country” man: a non-Traveller man.
“Halting site”: purpose-built residential accommodation for Travellers provided by a local municipal authority.
“Pavee”: another word for Traveller.
“Settled”: non-Traveller.

## Findings

Three themes were generated through the data analysis process: (a) Theme 1: Key determinants of Traveller men’s mental health, (b) Theme 2: Contemporary Traveller masculinities, and (c) Theme 3: Navigating support seeking and coping with distress.

### Theme 1: Key Determinants of Traveller Men’s Mental Health

#### Structural Barriers

Participants reflected upon a range of structural barriers, most notably accommodation/homelessness, education, and unemployment. These barriers were inextricably linked and impacted not just in terms of social disadvantage and marginalization, but more fundamentally in terms of identity, active citizenship, as well as having ripple effects on Traveller men’s mental health.

You’re on the dole, she’s [wife] on the dole. You can’t provide. Now you might have other families there that’s doing well for themselves. And that puts you under pressure. And you’re trying to get out and you can’t get work. So, things like that, it does affect you. (James)

It’s where you’re living as well, if you’re not feeling the best and you need to get away for an hour [but] you’re stuck in a small caravan with three or four kids all on top of one another, especially on a rainy day. (Benny)

Participants’ accommodation ranged from private rental or standard accommodation provided by the Local Authority, to Traveller-specific accommodation such as halting sites or group housing schemes, to emergency accommodation (homelessness). While halting sites facilitated the preservation of Traveller culture through social connectedness to family and community, the reality was that local housing authorities were seen as reneging on their responsibilities by providing substandard, crowded and claustrophobic living conditions, and lack of green spaces. For some, halting sites represented a double-edged sword not only by being a potential resource for positive mental health but also by compounding psychological distress.

You wouldn’t put a dog in them [halting-site] and yet humans are expected to live in these conditions? It’s a good thing living on a halting site, but it’s also a bad thing. When you’re up, you’re up, when you’re down, you’re down. (Robert)

These substandard conditions reinforced feelings of neglect and ambivalence from local authorities. Eamon noted how living in homeless accommodation stood in stark contrast to what he had expected for his family and how this was a major contributing factor to his psychological distress.

It gets me down. My child has been in the homeless since he was born . . . you’re pushed back, it’s like you are not wanted. I never thought I would be homeless in my life. When I got married, I thought I would have a house, but 3 ½ years in the homeless is a long time. (Eamon)

Participants reflected upon the challenges of living in mainstream accommodation and not fitting in within the non-Traveller community; of swapping the camaraderie and freedom of the halting site for the more sterile and closed environment of mainstream accommodation; of feeling like an outsider and living under a constant cloud of suspicion and prejudice—as the de facto fall guys for any deviant or antisocial behavior that might occur.

Where I grew up in a Travellers halting site, everyone’s a Traveller. So everybody knows each other’s mood. But your comfort zone is completely taken away from you when you’re in a settled [mainstream] community. You’re outside, you’re a Traveller, and with that comes the stories. He’s going to be messing, he’s going to be drinking, he’s going to be bringing a crowd . . . all the antisocial behavior . . . (PJ)

Lower educational attainment and early-school leaving emerged as a key structural factor that impacted on Traveller men’s lives. Participants recalled childhood memories of having faced both name-calling and other forms of racism and discrimination from peers and teachers alike. Francis recounts being subject to a more insidious form of constant ridicule within the classroom as well as more violent forms of abuse outside the classroom.

[In school] if you couldn’t pronounce the word properly you would be made fun of and you would be embarrassed to say that word again . . . I remember coming back one day from school and we met a group of lads and one of them shoved my face into horseshit. (Francis)

In a similar vein, Derek describes the prejudicial and unjust standards of school discipline that he faced as a Traveller child, and how being under constant surveillance for the so-called deviant behavior within the school environment resulted in negative reinforcements of his ethnic identity.

. . . yet when we were brought into the principal’s office, I was suspended whereas you [a non-Traveller] were given 500 lines . . . and a lot of my brothers and cousins experienced that as well . . . you were just under a lens all the time. (Derek)

Some participants recounted impediments to pursuing an education from within their own community. Robert’s recollection of being labeled “a country [non-Traveller] boy” for attending school conveys the toxicity of how some past generations of Travellers experienced the education system and the subsequent apathy they felt toward formal education.

I’d be going to school, and they would stop me at the entrance to the site and mock me: “Go away now you country [non-Traveller] boy . . . school will get you nowhere.” (Robert)

It should be acknowledged that several participants described a more recent sea change regarding school attendance and attitudes to education, with increasing numbers of Travellers completing second-level education and going on to the third level.

There’s a lot of Travellers sticking in school . . . going on to college and getting jobs. So, it’s starting to change. (Eamon)

An inevitable offshoot of a poor educational experience and early-school dropout was that Travellers faced additional barriers when attempting to secure employment, especially in professional careers. This, coupled with what were regarded as more deep-rooted and systemic discrimination and racism within employment institutions, rendered Travellers’s attempts to seek employment of any kind extremely challenging. The pragmatic course of action for some was to conceal their ethnic identity or assume the persona of a non-Traveller person.

It’s never heard of that a Traveller is going to college . . . you’ve never heard of a Traveller doctor or a Traveller guard. (Eamon)

. . . if I walk and talk and dress like a country man (non-Traveller), then I have a better chance of getting the job. (Charlie)

The significant and intersecting barriers that Travellers faced in attempting to secure employment resulted in unemployment for many, with the likelihood of securing any future employment representing a bleak prospect.

You can’t get a job and you are dependent on social welfare; a lot of men my age are struggling. (Francis)

While participants described a number of far-reaching and damaging effects of unemployment, it was the impact on Traveller identity and self-esteem that was singled out for most attention. Against a strong tradition of skilled craftsmanship within the Traveller community, participants cited how being unemployed left them bereft of skills and emasculated, resulted in a lack of structure and routine to their daily lives, and forced them to face the weekly ignominy of the social welfare office.

Unemployment is a big thing . . . it’s the lack of skills. Like Travellers are very hands-on guys, even when they’re younger. (PJ)

. . . when I wasn’t working it was like you were going to bed, you’re getting up, it was the same thing day in and day out. And it was mentally tough. (Derek)

You go to the social welfare, the majority you see there is Travellers . . . collecting their money. It will always be Travellers down there collecting their money. (PJ)

#### Cultural Barriers

Compounding the impact of these structural factors on Traveller men’s lives was a perception of tokenism and inertia on the part of state agencies to effect any real change. There was an air of pessimism and disillusionment among participants, many of whom felt invisible because of not having their needs met by the State and by a perception that nothing was going to change. James’s sense of frustration at this failure at a systems level to fully understand and empathize with the real-world challenges impacting Travellers’s lives was echoed by many.

Yes, it’s absolutely brilliant that we are recognized as an ethnic group . . . but there’s not a whole lot of change happening on the ground for Travellers and that pisses me off . . . What I find sometimes with [names external] organizations . . . they go out, they take a couple of photos, do a bit of research, and you’ll never see them again . . . they [non-Travellers] don’t really see Travellers. (James)

More broadly, participants described a range of other life experiences in which they were subject to prejudice, discrimination, and racism and which significantly affected their mental health (“that is the hardest thing—the exclusion,” Tommy). This ranged on a continuum from more blatant and institutionalized forms of such behavior, to more subtle but nevertheless insidious and harmful examples in routine day-to-day social contexts. Among the more common forms of such behavior were derogatory name-calling, being refused entry to social and recreational spaces, and living under a constant cloud of suspicion. James sums up how structural barriers and the day-to-day impediments he faces in accessing recreational spaces and living under a constant glare of suspicion and accusation impact his mental health.

You can’t go to the pub, you know they are not letting you in because you are a Traveller. You can’t get a job, because you are a Traveller. You can’t go to a shopping center without getting the eyes and being followed. You can’t get accommodation because you are a Traveller. That all affects you mentally. (James)

In Jack’s case, this gravitates toward paranoia and internalized racism, as he becomes fixated upon his ethnicity being the constant focus of negative and derogatory attention.

As a Traveller, it’s always at the back of your head. I could be sitting down there yapping with you and two people could be over there laughing and having a joke and for a split second I think they are laughing at me. (Jack)

This notion of being different, of being “other” than mainstream society, became deeply ingrained in how participants constructed their identity. More worryingly, there was a degree of acceptance and inevitability about the multiple layers of discrimination and racism which, as Francis points out, becomes “the norm” for Travellers. However, later in the interview, Francis acknowledges that this came with a heavy price in terms of Travellers’s sense of self-worth and mental health.

The sad thing is when you face racism 7-days a week since the day you’re born, you come to expect it, you think nothing is going to change . . . there are people out there that just put you in a box and that’s where you’re going to stay . . . it’s the negativity associated with Travellers that just leaves them feeling absolutely worthless. (Francis)

These incessant and layered forms of discrimination and racism inevitably led to Travellers becoming more circumspect in their interactions with and fundamental trust of non-Travellers. Inevitably, it was felt that Travellers tend to gravitate toward their own community for solidarity and support and to adopt a more insular view of the world.

. . . in most of the Traveller community, a Traveller sticks to a Traveller. They do not cross boundaries and they don’t go out towards the settled [non-Traveller] community . . . so, for example, like you’re playing football, you go on the Traveller football team, that’s all you do. (PJ)

You’ll find loyalty in Travellers, yeah I would live nowhere else like. (Jack)

Although not specifically attributed to the structural barriers that Travellers faced, a number of participants described the loss of children and/or loved ones as having a significant impact on their mental health.

I buried a baby—I’ve been put through the mill. (Robert)

** years old, she died in my arms over that lifestyle [reference to drug habit]. That left me depressed, I wanted to join her. (Paul)

Exposure to death by suicide was a significant contributing factor to psychological distress (“. . . in this particular town the morale is so low because of suicide,” Robert). There was broad consensus that the disproportionately high suicide rate among Traveller men in Ireland was, as Francis describes, underpinned by a deep-rooted sense of hopelessness and despair, emanating from the entrenched structural and cultural barriers that Travellers faced.

If you can’t see a way out, and you think every day is gonna be the same until the day I die you start to think what’s the point? What’s the point of living? Every day is a struggle, for work, for accommodation, just for the simple things in life. (Francis)

James attributes his suicidal behavior to the financial loss that precipitated more significant loss of reputation and standing within his community.

Because he (male relative) went from having everything to having nothing. And this is why he is trying to kill himself. And in time he will. I’m telling you he has already said it now that he doesn’t want to live . . . “I want to go down to my Daddy.” (James)

### Theme 2: Contemporary Traveller Masculinities

#### Defining Characteristics of Traveller Masculinity

The construction of Traveller masculinities in this study was strongly influenced by a patrilineal tradition as well as by historical and ongoing tensions related to structural inequalities, racism, prejudice, and discrimination in the face of Travellers’s efforts to preserve their ethnic identity. As a minority ethnic group, there was a strong commitment to honor and pass on traditional Traveller values and customs by outwardly displaying an allegiance to kinship and collegiate spirit within the wider Traveller community. In the face of considerable adversity, being stoic, self-reliant, and not showing vulnerability emerged as important markers of Traveller masculinity. Indeed, showing vulnerability was, as Charlie and James note, seen as compounding psychological stress.

It’s that persona of Traveller men that they can’t be perceived as weak and always have to be strong. And the weaker you are, I suppose, the more vulnerable you are. As the head of the family people look up to them [men], not them looking up to somebody else. (Charlie)

Masculinity, showing your weakness. Imagine crying in front of another man? A Traveller man. You’d be the talk of the place . . . you’re not allowed to cry. I’d hide an awful lot from them (family). It’s an awful lot of pressure. (James)

The embodiment of physical strength and toughness was reported as an important marker of Traveller masculinity. In Paul’s case, the pressure to uphold this pillar of masculine identity was exacerbated by having a physical disability, even though being “this hard man” was not consistent with the identity to which he aspired and caused him significant distress.

I grew up with [physical disability] so I felt weaker than everyone else . . . thinking I’d not be able to fit it. I have a big heart, I want to get on with people. But I thought I had to act this hard man image, I was always going around fighting . . . I ended up doing [x] years in prison. All because of an image that I didn’t want to be. It used to fill me with anger, anxiety and depression. (Paul)

Some younger participants challenged this more traditional construction of Traveller masculinities by positioning themselves as being more open to reconfiguring a different type of Traveller masculinity to past generations.

They (older Traveller men) are more of a quiet generation . . . they will just close up, that’s what they will do. They will keep it in. (PJ)

It’s just a stupid mentality that was passed down. (Robert)

The strong sense of close-knit community that came with living in close proximity to extended family members was framed as an important aspect of Travellers’s masculine identity, bound together by social and emotional connectedness, as an extended family.

The island of Ireland is a very small island, but so connected within the Travellers. Like everyone would know such a person like, such and such came from such a family. All families would be somehow intertwined together . . . it’s like a big jigsaw. (PJ)

#### Key Milestones in the Transition to Manhood Within Traveller Community

A number of key benchmarks or milestones were evident in the transition to manhood within the Traveller community. These included attaining financial autonomy, having material possessions, getting married (within the context of heteronormative relationships), becoming a father, and facing up to expectations and obligations associated with being a provider and protector of one’s family. Marriage, in particular, was seen as a particularly important life transition, bestowing a responsibility and pressure to provide, to get a job, become a father, and become head of one’s own household.

I got married at 18, you’re expected to have a child straightaway. That’s one of the things as a Traveller, young boy or girl, rearing children . . . going out and getting money to provide for your family. And you have to have stuff, to show stuff . . . the good car, the good trailer, the good mobile. (James)

Findings suggest that these highly gendered role expectations were ingrained from a young age and passed on from one generation to the next.

Getting a job, getting money, feeding the family, getting the kids clothes. (Eamon)

Meeting these key Traveller masculinity milestones conferred status among one’s peers, while failing to do so risked being consigned to a subordinate or marginalized status. Paradoxically, the considerable structural barriers facing Traveller men prevented them from reaching many of these milestones. In James’s case, the prospect of “failure” in terms of not matching up to these landmarks and role expectations cascaded beyond him “failing” his family to his family also being labeled a “failure.”

By you not having money, and you not having a car, and you not being able to provide for your family, other Travellers look down on you, other Travellers disrespect you . . . If you’re a failure, your family’s a failure, you’ve let down your family. (James)

Against this weight of expectation that came with being a Traveller “man,” Charlie makes a somewhat pragmatic assessment to shield his wife from his worries so as not to be a burden.

If something was worrying me and I tell my wife, then she is worrying about it. So let alone it will cause me stress, it is causing somebody else stress. (Charlie)

The pressure to make these transitions prompted many young Traveller men to forego potential life opportunities and to assume adult responsibilities at a comparatively young age. PJ described choosing not to opt for further study or travel after secondary school because of the expectation to “put your life on hold” and instead to marry and start a family. In the context of participants in this study, marriage, relationships, and sexuality within the Traveller community were framed within a heteronormative lens. Francis draws upon a rather poignant analogy between there being only one way to “get out” of a halting site and the pressure on gay Traveller men not to “come out.” Rather, the expectation was to “act normal” within the boundaries of heterosexual relationships (and heterosexual marriage).

If you live in a halting site, you are most likely living in a cul-de-sac. There is only one way in and one way out. So, if you are bisexual or gay or trans, it can be very hard to get out. That bad feeling is going to be there until they get married and “act normal.” . . . I think sexuality has got to do with some of the suicides and that expectation to be “the norm.” (Francis)

### Theme 3: Navigating Support Seeking and Coping With Distress

#### Navigating Support Seeking

Participants approach to help-seeking during times of psychological distress fell into two polarized categories: those mostly older participants who were resistant to seeking help and those mostly younger participants who were much more proactive about seeking help. The former aligned with prevailing Traveller masculinity norms that were prominent in this study—as the patriarchal figure in the household, seeking help had connotations of “being ‘weak’” (Jack), “not being able to cope” (Francis), and was abhorrent to the more manly roles of providing for and protecting one’s family. It was also incumbent on Traveller men to set aside their own needs in favor of their families’ needs. The very acknowledgment of being “mentally unwell” ran the risk of “bringing shame” (Eamon) or being a “burden” (Charlie) on one’s family.

. . . so there’s the whole stigma that if I show weakness, or I show that I’m not mentally well, would I be treated differently within the community . . . would I be judged and am I bringing shame on the family? (Eamon)

Why would I lay my burdens on them? And let them sink down. (Charlie)

Older participants, in particular, found it difficult to talk about suicide and highlighted the intense mental health stigma in their community. Finding the right language that transcended guilt, shame, stigma, and fear was both challenging and acted as a barrier to help-seeking. There were concerns that talking about suicide might prompt further suicidal behavior and that suicide cast a shadow on the bereaved family’s reputation and standing within the Traveller community.

There is a saying in our community that you are bad with your nerves. It’s [suicide] not talked about, it’s a taboo subject . . . Fear of mentioning the word suicide in our community can be enough to keep people isolated and in fear of looking for help. (Francis)

. . . they [Traveller men] wouldn’t want it brought up in conversation in case others might do the same thing. (Charlie)

. . . it [suicide] is kind of insulting to the family members that they leave behind. That’s the way Travellers are . . . you’re saying they haven’t got the resilience, that they weren’t tough enough, that they were a coward? (Eddie)

Approximately half of mostly younger participants in this study adopted a more open and proactive approach to mental health and help-seeking. This represented a generational shift, whereby younger participants contested the pretense of a more stoic and self-reliant embodiment of Traveller masculinity in favor of an increased openness to acknowledging fluctuations in mental health and to normalizing help-seeking during times of psychological distress. For others, it represented a desire to shed the pretense of “the hard man” and learning to be more accepting of oneself.

It’s only now that the younger Travellers are coming along and saying: ‘We’ll talk about it (mental health).’ . . . we are not as thick [stubborn] about it, it’s like everybody goes through it. (Robert)

So just accept that you need the help and it’s okay to ask for it, stop pretending that you are something that you are not. (Eddie)

Despite experiencing a significant amount of stigma in relation to mental health, several participants noted that, with the right conditions, Traveller men can be very open to discussing their mental health. There was a clear preference for more informal and flexible supports over mainstream professional services, as well as using action-orientated approaches and activities that appealed to Traveller men, such as football or boxing, to initiate and strengthen conversations around mental health.

You create the right conditions, get them in the right place, Traveller men will open up . . . You run football groups, fitness groups. (James)

Peer-led Traveller initiatives were seen as creating a sense of trust and conferring credibility to a particular program, while having someone to take the first step in opening conversations about mental health was also important.

It’s that trust in another Traveller . . . you don’t have to tell this story 10 times, he totally understands what’s going on for me. (Derek)

Participants felt their engagement with statutory services could benefit greatly from staff undertaking anti-racism training. They made particular reference to the embarrassment that Travellers with poor literacy felt when asked to complete a written form, which acted as a deterrent for some Traveller men accessing services.

Services are not culturally aware . . . they need training. You go to make an appointment, the first thing you are given is a form and a pen—“fill this out.” A lot of Traveller men might not be able to read and write and the whole waiting room is full of people, you’d be so embarrassed. (Francis)

Participants highlighted the need to embed Traveller culture across programs and statutory services and to employ more Travellers to deliver such initiatives. There was strong support for bridging statutory services through Traveller-specific organizations and/or providing Traveller-specific counseling services to engage Traveller men around their mental health. For example, Derek highlighted the value of a Traveller-specific text line (“text Pavee”) for Traveller mental health.

. . . so now the service provider knows it’s a Traveller speaking to them. (Derek)

#### Coping With Distress

Participants reflected on both positive and negative coping strategies in their endeavors to build resilience and manage their mental health. Family was at the heart of what Traveller men in this study valued and lived for. Fulfilling one’s responsibilities as a father, as well as nurturing and being nurtured by family, was repeatedly referenced as what grounded and fulfilled Traveller men. There was an acknowledgment of the role played by the wider Traveller community “family” in terms of staying connected and accessing support when faced with challenges.

What makes me feel good is being around my family, getting together, doing things with my family. (Eamon)

I wanted to join her (deceased wife). But then I realized I have x [names number of] children, I can’t leave them behind. So, I turned my whole life around. (Paul)

The thing about Travellers is when something happens. It brings people an awful lot closer together . . . it helps having people around you. You’re not sitting there all by yourself getting bad thoughts. (Robert)

There was broad consensus among participants about the value of physical activity and sport as an enabler of health and well-being. Many referred to its cathartic effect in terms of releasing frustrations and dealing with stress. For those who were unemployed and whose daily routines were not defined by paid employment, physical activity and sport offered some respite in terms of providing a purpose, structure, and routine to daily life, offering a social outlet, and an alternative to ruminating on problems.

The best thing, to be honest with you, if you have bad days, is the comfort of a gym. Physical exercise, a boxing bag. Just take all your frustrations out on that. (PJ)

There was some evidence of participants finding solace in religious beliefs and practices. Paul identifies his faith and his engagement in regular religious rituals and practices, as an important source of inspiration and support in sustaining his recovery from drug addiction.

God is definitely on my side anyway. There is definitely someone watching over me . . . What I do now is I go to churches, I light a candle, I’ll say a prayer, kiss a statue or bless me face . . . it keeps me going and keeps me sane. (Paul)

Findings also revealed more negative coping strategies, some of which revolved around more restrictive Traveller masculinity norms, such as concealing vulnerability from loved ones (see Theme 1). Participants also highlighted alcohol consumption and drug use as “coping” strategies used by some Traveller men to deal with psychological distress and were mindful of the potential ramifications for further mental health issues.

Get to the pub, a couple of pints, that’s your worries gone for a couple of hours. (Jack)

. . . some Travellers feel like they need to put up this image . . . if they go into a party . . . there is all the other Travellers in there, drinking and sniffing, it’s the whole thing like now . . . build their confidence . . . they are not thinking of the comedown, the depression that comes with it. (Paul)

## Discussion

The aim of this study was to explore the lived mental health experiences of Traveller men affected by suicide in Ireland. The study adopted an intersectional approach that explored the simultaneous interactions of gender and ethnicity with other social categories to capture the multiple layers of disadvantage and blended identities of Traveller men. The impetus for the study was prompted by particularly high suicide rates among male Travellers in Ireland and by previous studies which document significant social disadvantage, social exclusion, and discrimination in the Traveller community (AITHS, 2012).

The first research question sought to determine the key sources of psychological distress and how they intersect among Traveller men affected by suicide in Ireland. Participants reflected upon a range of structural and cultural barriers that fundamentally affected their lives. In relation to accommodation, these challenges ranged from homelessness, to substandard, crowded and claustrophobic living conditions in halting sites, to being subjected to prejudice, marginalization, and feeling like an outsider within a mainstreamed non-Traveller accommodation context. This was compounded by a sense of inertia on the part of local housing authorities to address Travellers’s concerns in relation to accommodation and living conditions. Systemic racism, prejudice, and discrimination were implicated in early-school leaving and manifested both in terms of more insidious within-classroom abuse and more violent forms of abuse outside the classroom. Social stress theory provides insights into the links between discrimination and suicidal behavior and may be particularly relevant for Traveller men. Social stress originates from the absence of resources to meet one’s goals in life or to maintain one’s current level of social functioning (Cohen & Wills, 1985). Exposure to multiple and chronic stressors can strain an individual’s adaptive capacities and result in mental health disorders and suicidal behavior (WHO, 2014). Yur’yev et al. (2013) concluded that social exclusion and discrimination is a strong example of one such chronic stressor. The wider literature has reported that rejection, discrimination, and stigmatization of subpopulation groups can evoke suicidal behavior (McKey et al., 2022; WHO, 2014).

Findings suggest that the pressure to transition to key Traveller milestones (marriage, becoming a father) prompted many young Traveller men to forego further education and to assume adult responsibilities at a comparatively young age. However, the structural barriers that Traveller men faced often impeded them from providing the life or resources that they wished for their families which resulted in a sense of “failure.” These findings are notable in the context of the Integrated Motivational-Volitional (IMV) model of suicidal behavior, which suggests that experiences of defeat (failed struggle, powerlessness, losing social status) and negative social comparisons can lead to feelings of entrapment and suicidal behavior (R. C. O’Connor & Kirtley, 2018; Wetherall et al., 2019). Moreover, it is proposed that sensitivity to defeat may be increased by socially prescribed perfectionism—an unrealistic outlook on what we believe others expect of us (D. B. O’Connor et al., 2007). Thus, in the context of Traveller men’s “failure” to achieve expectations or benchmarks associated with being a Traveller man, it becomes clear how intersecting aspects of identity (gender, age, ethnicity) can create multiplicative effects on suicide risk among this population. These findings are consistent with previous studies which demonstrate that Irish Travellers experience significant social disadvantage, social exclusion, and discrimination, which have significant knock-on repercussions for their mental health (AITHS, 2012; Villani & Barry, 2021; Watson et al., 2017).

Against a backdrop of a proud tradition of skilled craftsmanship within the Traveller community, unemployment left many Traveller men in this study feeling emasculated and rudderless, and dependent on state support services in which they had little confidence in ever effecting any real change. Unemployment has been reported to have a causal effect on suicide with a stronger relationship for men compared with women (Guntuku et al., 2021; Platt, 2011; Qin et al., 2000). Traditional expectations about the male provider and breadwinner roles appeared to be intensified among Traveller men in this study, with unemployment being associated with a distinct loss of role and purpose. Failing to live up to the provider role resulted in a dislocation of participants’ perceived role of breadwinner in the household and to profound feelings of shame, embarrassment, and emasculation for many men. At the same time, participants described feeling pressurized to remain stoic, strong, and silent in dealing with what were frequently formidable challenges in living up to such responsibilities. Loss of tradition and culture has previously been identified to disrupt Traveller men’s sense of identity, leading to feelings of powerlessness and increased risk of suicide (Hodwitz & Frey, 2016; O’Donnell & Richardson, 2020). This loss of tradition and culture links to the suicide risk factor of “perfectionistic self-preservation”—self-promotion and the concealment of “errors” to present a specific image—as described by Besser et al. (2010).

Building on the findings from previous studies (AITHS, 2012; Gmelch & Gmelch, 2014), there were many other more routine life experiences in which participants described being subject to prejudice, discrimination, and racism. These included derogatory name-calling, being refused entry to social and recreational spaces, being subjected to physical violence, along with living under a constant spotlight of suspicion and accusation that prompted manifestations of internalized racism. For many, there was a tacit acceptance that these multiple layers of discrimination and racism were inevitable or “the norm” for Travellers, leaving them with a very negative sense of self-worth and feeling like second-class citizens. This rejection subsequently led to feelings of reduced self-efficacy and hopelessness which have been identified as key suicide risk factors (WHO, 2014). These findings resonate with previous studies reporting that prolonged exposure to discrimination had ripple negative effects on Travellers’s self-esteem and self-worth and prompted feelings of hopelessness and disconnection (O’Donnell & Richardson, 2020; Villani & Barry, 2021). They add further weight to the description of Travellers by Hayes (2006) as the “other” of Irish ethnic identity and resonate with Durkheim’s theory of anomie. Participants described becoming more circumspect in their interactions with and trust of non-Travellers, and instead gravitated more toward the solidarity and support of their own community. This is in keeping with the notion of a collective masculine identity (Connell, 1995), whereby the wider Traveller community was seen as an extended family, bound together, in particular, by core masculine values and by the social and emotional connectedness and solidarity cultivated by communal living in Traveller sites.

The second research question sought to explore the barriers and enabling factors to Traveller men seeking help and accessing support during times of psychological distress. A key backdrop to this is how Traveller masculinities in this study were rooted in a patrilineal tradition and underpinned by structural inequalities as well as historical and ongoing tensions related to systemic racism, prejudice, and discrimination (AITHS, 2010). Among older participants, in particular, the sense of compulsion to preserve and demonstrate public allegiance to Traveller values and customs was very evident, while being stoic, self-reliant, not showing vulnerability and the embodiment of physical strength and toughness emerged as important markers of Traveller masculinity—findings which are consistent with previous studies (Cush et al., 2020; O’Donnell & Richardson, 2020). Also important in this regard was the emergence of key benchmarks or milestones in the transition to manhood within the Traveller community—most notably attaining financial autonomy, having material possessions, getting married (within the context of heteronormative relationships), becoming a father, and facing up to expectations and obligations associated with being a provider and protector of one’s family. Not meeting these milestones risked being labeled a “failure.” Against this weight of expectation that came with being a Traveller “man,” some participants reflected on the pressure they felt under to conceal “stress” so as to avoid being seen as “weak” or “unable ‘to cope’.” There were concerns about not wanting to be “a burden” or to “bring shame” on one’s family, findings which resonate with previous findings (McKey et al., 2022) and with the interpersonal theory of suicide (Joiner et al., 2009). The strong associations between mental health and stigma permeated into difficulties in relation to talking about suicide and finding the right language that transcended guilt, shame, and fear. Part of the grieving process for those bereaved by suicide was coming to terms with the shadow cast by a suicide on the family’s reputation and standing within the Traveller community. In grappling with this implicit stigma that was associated with mental health/suicide, conversations about suicide inevitably cascaded into feelings of confusion, helplessness, and despair. Links between stigma and mental health are well established among Traveller men (Hodgins & Fox, 2014; McKey et al., 2022; Villani & Barry, 2021) and have been reported to inhibit help-seeking in relation to mental health difficulties (AITHS, 2010; O’Donnell & Richardson, 2020; Keohane & Richardson, 2017).

A positive finding from the study was the more open and proactive approach to mental health and help-seeking adopted particularly by younger participants, and which represented a normalizing of help-seeking during times of psychological distress. There were practical suggestions of how to engage Traveller men, including the prioritization of more informal supports and settings over mainstream professional services, using action-orientated activities (e.g., football or boxing) to initiate and strengthen conversations around mental health, as well as adopting a flexible approach with a focus on peer support. Against a backdrop of Travellers’s experiences of discrimination in accessing health services (McFadden et al., 2018), there was strong support for service and program providers undertaking anti-racism training, for more peer-led and Traveller-specific programs, and for bridging statutory services through Traveller-specific organizations. For example, similar to findings from Brack and Monaghan (2007), illiteracy was highlighted in this study as a considerable barrier for Traveller men in seeking support for mental health issues. Mental health literacy is also important in terms of fostering more positive attitudes to mental health (Lee et al., 2020) and is positively correlated with help-seeking (Gorczynski et al., 2017). Notably, the call for increased resourcing of Traveller-specific counseling services comes 13 years after a similar call (AITHS, 2010). In seeking to build resilience and manage their mental health, participants emphasized the support of family and community, having a purpose, structure, and routine to one’s life, and finding solace from religion. Conversely, maladaptive coping mechanisms included alcohol and drug misuse, and adhering to more restrictive Traveller masculinity norms, such as concealing vulnerability from loved ones.

## Conclusion

This study sheds new light on the factors underpinning the high suicide rate among Traveller men in Ireland. It does so by applying an intersectional lens to unravel how the lived experiences of Traveller men affected by suicide are shaped by intersecting structural factors and social identities, which result in Traveller men being exposed to multiple and intersecting layers of disadvantage, racism, marginalization, and discrimination. The intersection of this potent mix of structural inequalities, internalized racism, Traveller masculinities, and strong historical associations between stigma and mental health/suicide within the Traveller community lies at the heart of the heavy burden of suicide carried by Traveller men and poses significant barriers to Traveller men accessing support during times of psychological distress. Findings offer some practical suggestions of positive coping strategies used by Traveller men during times of psychological distress as well as how to engage Traveller men in their mental health. In addition to informing the design of a wider therapeutic mental health DST intervention targeted at Traveller men, findings have the potential to inform the development of more gender- and culturally appropriate interventions to support suicide prevention efforts aimed at this priority group. From a broader policy perspective, findings reinforce the need for early and targeted Traveller education and training interventions, for increased resourcing for Traveller accommodation, and for more concerted efforts to deal with discriminatory behavior toward Travellers at a societal level. Future research should focus on pathways to recovery among Traveller men with direct past experience of suicide behavior, with a view to informing the design of mental health and suicide prevention supports for Traveller men.

## Limitations

While the study sought to include the voices of a range of Traveller men affected by suicide, the findings cannot purport to be inclusive or “representative” of all Traveller men. Caution is warranted in drawing inferences between this study’s findings and causal risk factors for suicidal behavior. Rather, the focus was on drawing out the lived experiences of Traveller men affected by suicide to elucidate the complexity of Traveller suicide. The study would have been enriched by the inclusion of more participants who had direct experience of suicide and suicidal behavior. However, recruiting such participants proved to be a considerable challenge in practice. While all participants were assured of confidentiality and invited to speak candidly, it is possible that some may have been guarded in sharing personal experiences out of fear of compromising family members or other members of the Traveller community.

## Notes

1. Postprimary state examination.
